# Effect of Threshold Inspiratory Muscle Training on Functional Fitness and Respiratory Muscle Strength Compared to Incentive Spirometry in Children and Adolescents With Obesity: A Randomized Controlled Trial

**DOI:** 10.3389/fped.2022.942076

**Published:** 2022-07-07

**Authors:** Phatthareeda Kaeotawee, Kanokporn Udomittipong, Akarin Nimmannit, Prakarn Tovichien, Apinya Palamit, Pawinee Charoensitisup, Khunphon Mahoran

**Affiliations:** ^1^Division of Pulmonology, Department of Pediatrics, Faculty of Medicine Siriraj Hospital, Mahidol University, Bangkok, Thailand; ^2^Research Department, Faculty of Medicine Siriraj Hospital, Mahidol University, Bangkok, Thailand

**Keywords:** effect, threshold inspiratory muscle training, respiratory muscle strength, obese children and adolescents, incentive spirometer, 6-MWT, obesity

## Abstract

**Background:**

To determine the effect of threshold inspiratory muscle training (IMT) on functional fitness and respiratory muscle strength (RMS) compared to incentive spirometry (IS) in children/adolescents with obesity.

**Methods:**

A total of 60 obese children/adolescents aged 8–15 years were randomized into the threshold IMT group (*n* = 20), the IS group (*n* = 20), or the control group (*n* = 20). The IMT group performed 30 inspiratory breaths with the intensity set at 40% of baseline maximal inspiratory pressure (MIP) twice daily for 8 weeks; the IS group performed 30 breaths with sustained maximum inspiration twice daily for 8 weeks; and, the control group was assigned no training device for 8 weeks. Six-min walk test (6-MWT), RMS, and spirometry were compared between baseline and 8 weeks.

**Results:**

Six-MWT distance (528.5 ± 36.2 vs. 561.5 ± 35.2 m, *p* = 0.002) and MIP (121.2 ± 26.8 vs. 135.3 ± 32.1%Predicted, *p* = 0.03) were significantly improved after 8 weeks of IMT training. There was no significant difference in any evaluated pulmonary function parameters between baseline and 8 weeks in the IS or control groups; however, 6-MWT distance demonstrated a trend toward significant improvement in the IS group (526.9 ± 59.1 vs.549.0 ± 50.6 m, *p* = 0.10). No significant difference among groups was found for any variable relative to change from baseline to post-training.

**Conclusion:**

Eight weeks of threshold IMT training significantly improved both inspiratory muscle strength (MIP) and functional fitness (6-MWT) in children/adolescents with obesity. Eight weeks of IS training yielded a trend toward significantly improved functional fitness.

## Introduction

Obesity is a major public health problem that affects children, adolescents, and adults worldwide, and that exerts adverse impact on many systems, including the respiratory, cardiovascular, and metabolic systems (1–3). The effects of obesity on the respiratory system include altered pulmonary function and exercise intolerance, and comorbid obesity can also worsen both asthma and obstructive sleep apnea (4). Several studies reported reduced functional fitness and reduced respiratory muscle strength (RMS) in children and adolescents with obesity (5–9), and these deficiencies may contribute to dyspnea on exertion. The principle pillar of treatment for obesity is weight reduction. Nutritional control and exercise are the main components of a weight loss program; however, some obese individuals experience exercise intolerance and/or limited functional fitness (10, 11) – both of which are obstacles to weight reduction.

Inspiratory muscle training (IMT) helps to improve both RMS and functional exercise capacity. The devices used for IMT mainly include pressure-based and volume-based loading type devices (12). Threshold IMT is a pressure-based loading device, and is the most commonly used inspiratory muscle trainer for improving the strength and edurance of the respiratory muscles (13). Incentive spirometer (IS) is a volume-based loading type device. IS is frequently used to increase lung volume and to prevent pulmonary complications after thoracic or abdominal surgery, and it is also employed for IMT *via* a technique known as sustained maximum inspiration (14). The IS device is less expensive than the threshold IMT device.

Many studies of threshold IMT (15–17) and IS (18, 19) training among obese adults reported substantial improvement in 6-min walk test (6-MWT) distance, inspiratory muscle strength, and spirometry. In children and adolescents, studies of the effect of threshold IMT (20–27) and IS (28–30) on pulmonary function were conducted in other diseases, including neuromuscular disease (NMD), cerebral palsy (CP), asthma, and cystic fibrosis (CF). To our knowledge, no study has investigated the effect of threshold IMT and IS on functional fitness, RMS, and spirometry in children and adolescents with obesity. In addition, no studies have compared the effects of these two devices on functional fitness and RMS in obese children and adolescents.

Therefore, the primary aim of this study was to investigate the effect of threshold IMT on functional fitness as measured by 6-MWT compared to IS in children/adolescents with obesity. The secondary objective of this study was to compare the effects of these 2 devices on maximal inspiratory pressure (MIP), which is a biomarker for RMS, and forced expiratory volumes and flows. We hypothesized that after 8 weeks of training, IMT by threshold IMT or IS would have more benefit on 6-MWT distance than no device, and that the benefit of threshold IMT would be superior to that of IS. If these devices can improve functional fitness in this patient population, we will have research evidence to recommend them as an alternative therapy for enhancing exercise capacity, which would facilitate weight loss.

## Materials and Methods

### Study Protocol

This prospective randomized controlled trial (RCT) recruited children and adolescents aged 8–15 years who were diagnosed with obesity, which was defined as a body mass index (BMI) *z*-score ≥ 2 according to World Health Organization (WHO) reference criteria (31). Patients with a history of neuromuscular, cardiac, or pulmonary disease; history of smoking or environmental tobacco smoke exposure; respiratory tract infection within the preceding 4 weeks; and/or, inability to perform pulmonary function testing (PFT) were excluded. Collected data included gender; age; height and obesity indices, including body weight (BW), BMI, BMI *z*-score, chest circumference (CC), waist circumference (WC), and WC/height (Ht).

Study participants were randomly allocated into 1 of 3 groups (IMT group, IS group, or control group) by block randomization. The IMT group received 8 weeks of at-home threshold IMT, and the IS group received 8 weeks of at-home incentive spirometer training. Functional fitness (6-MWT), RMS parameters [MIP, and maximal expiratory pressure (MEP)], and spirometry were evaluated at baseline before training and at 8 weeks after training by the same trained technician.

Participants in the threshold IMT group were instructed to perform IMT at an intensity of 40% of their baseline MIP using the threshold IMT device (Threshold IMT ^®^, Philips Respironics, Chichester, United Kingdom) with a resistance load of 9–41 cm H_2_O. Subjects were trained to position themselves in a sitting position in a chair, and to apply the provided nose clip to prevent airflow through the nose. They then exhaled completely, placed the mouthpiece of the device into their mouth, and then inhaled with maximal force to open the valve of the device. Subjects in the IS group were instructed to use the incentive spirometer device (Pulmo-gain ^®^, CA-MI, Langhirano (Parma), Italy) by performing a slow and deep inspiration until total lung capacity (TLC). Sustained maximum inspiration was set at approximately 3 s followed by expiration until achieving functional residual capacity (FRC). In both study device groups, a frequency of 3 sets of 10 breaths with a rest period of 1 min between each set was performed twice a day for 8 consecutive weeks. The control group did not receive any instruments for respiratory muscle training. Standard treatment for obesity, including recommendation for exercise, nutritionist consultation for dietary control, and evaluation of comorbidities, was given to study subjects in all 3 groups.

To improve the likelihood that study participants would adhere to their assigned training protocol (if they were in either the threshold IMT or IS groups), subjects were asked to complete a daily log indicating the date and time of their respiratory muscle training sessions. All participants were contacted weekly by the study assessment technician to ensure proper device use technique, to inquire about adverse events during interventions, and to inquire about adherence to the requested frequency of training.

This study was conducted at the Division of Pulmonology of the Department of Pediatrics, Faculty of Medicine Siriraj Hospital, Mahidol University, Bangkok, Thailand during October 2021–March 2022. The study protocol was approved by our center’s Institutional Review Board (IRB) (approval no. Si 277/2021), and was registered in the Thai Clinical Trials Registry (TCTR) (TCTR20211124001). Informed consent or assent (when applicable) was obtained from study participants and/or their legal guardian(s) before study enrollment.

### Anthropometric Evaluation

A standard scale (TANITA Corporation, Tokyo, Japan) was used to determine BW and height. BMI was calculated as body weight (kg) divided by height squared (m^2^). BMI was also expressed as *z*-score (BMI *z*-score) adjusted for gender and age according to WHO growth reference (32, 33). CC was measured at nipple level, and WC was measured between the inferior margin of the last rib and the iliac crest. The WC (cm)/height (cm) ratio was calculated.

### Spirometry

Spirometry was performed using a Vyntus™ BODY instrument (Vyaire Medical, Mettawa, IL, United States) according to American Thoracic Society (ATS)/European Respiratory Society (ERS) recommendations (34). FEV1, FVC, FEV1/FVC ratio, forced expiratory flow rate within 25–75% of vital capacity (FEF_25–75%_), and peak expiratory flow (PEF) were collected and recorded. All parameters except FEV1/FVC were reported as percentage of predicted value (%predicted) from multi-ethnic global lung function equations (2012) (35).

### Respiratory Muscle Strength

Inspiratory and expiratory muscle strength was assessed by MIP and MEP, respectively, using a Vyntus™ BODY instrument (Vyaire Medical) according to ATS/ERS guidelines (36). MIP was measured with the subject breathing in from residual volume (RV) to TLC, and MEP was measured during forced expiration from TLC to RV. The measurement considered for data analysis was the highest value among 3 acceptable maneuvers (without leakage and lasting for at least 1 s), and at least 2 were reproducible (not different more than 10% from the second highest value). A maximum of 9 maneuvers for each MIP and MEP assessment was performed, and both values were expressed as absolute and %predicted based on reference equations (37).

### 6-Min Walk Test

Functional fitness was assessed by 6-MWT (distance in meters), which is a recommended performance-based tool according to ATS/ERS guidelines (38). Participants were instructed to walk as fast as they could without running or jogging for 6 min on a flat, straight, there-and-back walking course. The course was 30 meters in length, and clearly visible cones were placed at each end of the course. Participants were given words of encouragement during the test, and the time remaining was announced at different timepoints during the test. The total distance covered by each participant in 6 min was recorded. Complaints of physical discomfort were also recorded. Participant heart rate, respiratory rate, blood pressure, and peripheral oxygen saturation (SpO_2_) were measured before and after the test. If a participant complained of heart palpitations, chest pain, or shortness of breath, the test was immediately stopped.

### Sample Size Determination

The sample size for this study was calculated using data from a pilot study that we conducted in 12 obese children and adolescents. That pilot study yielded mean 6-MWT distances of 540, 520, and 490 m (pooled standard deviation: 27 meters) in the IMT, IS, and control groups, respectively. Using a power of 0.8, a two-sided alpha level of 0.05, and an effect size 0.58, a minimum sample size of 20 participants per group was required.

### Statistical Analysis

SPSS Statistics for Windows version 18.0 (SPSS, Inc., Chicago, IL, United States) was the software used to analyze the data. The baseline characteristics of study participants are presented as mean plus/minus standard deviation for continuous data, and as number and percentage for categorical data. Paired *t*-test was used to analyze within group changes for normally distributed data. One-way analysis of variance (ANOVA) followed by Tukey’s *post hoc* test was used to compare continuous data among 3 groups, and chi-square test was used to compare categorical data among 3 groups. A *p*-value less than 0.05 was considered statistically significant for all tests.

## Results

**FIGURE 1:**
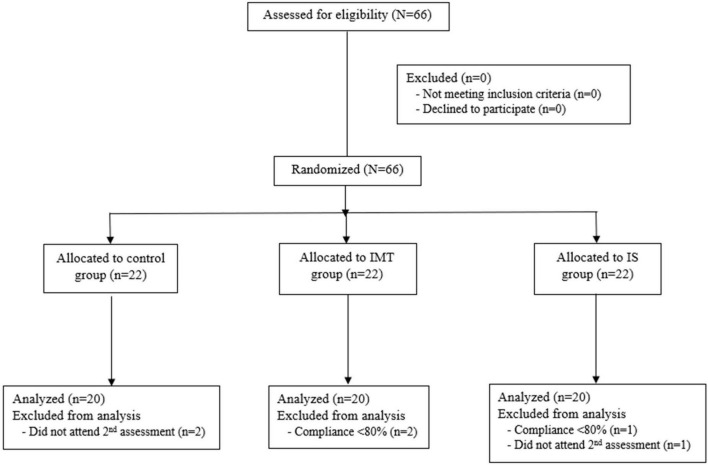
Flowchart of patient enrollment, group allocation, and data analysis protocol (IMT, threshold inspiratory muscle training; IS, incentive spirometry).

Of the 66 enrolled participants, 60 children and adolescents (20 participants per group) completed the 8 weeks of training with compliance over 80% and successful performance of all PFTs (Figure 1). The mean (±SD) age of control, IMT, and IS group subjects was 11.2 ± 2.55, 12.4 ± 1.92, and 12.5 ± 2.61 years, respectively. Age, gender, height, and obesity indices, including BW, BMI, BMI *z*-score, and CC, were statistically similar among the 3 groups at baseline; however, WC/Ht was significantly greater in the IMT group than in the IS or control groups (*p* = 0.01) (Table 1).

**TABLE 1:** Mean demographic data and obesity indices compared among the control, IMT, and IS groups.

	Mean ± SD			
Data	Control group (*n* = 20)	IMT group (*n* = 20)	IS group (*n* = 20)	*p*
Age (years)	11.2 ± 2.55	12.4 ± 1.92	12.5 ± 2.61	0.15
Male gender, *n* (%)	17 (85.0%)	13 (65.0%)	12 (60.0%)	0.19
Height (m)	1.51 ± 0.17	1.58 ± 0.1	1.57 ± 0.16	0.28
Body weight (kg)	73.8 ± 26.1	85.6 ± 22.6	78.4 ± 26.2	0.33
Body mass index (kg/m^2^)	31.3 ± 5.83	33.9 ± 5.47	30.9 ± 7.1	0.27
Body mass index *z*-score	3.56 ± 0.85	3.50 ± 0.71	2.99 ± 0.9	0.06
Chest circumference (cm)	95.5 ± 14.2	101.4 ± 14.8	95.2 ± 15.2	0.33
Waist circumference (cm)	100.9 ± 14.6	110.4 ± 13.5	99.5 ± 16.6	0.05
Waist circumference/height	0.67 ± 0.06	0.70 ± 0.06	0.63 ± 0.08	***0.01***

*A p-value < 0.05 indicates statistical significance. IMT, threshold inspiratory muscle training; IS, incentive spirometry. The bold italic means a p-value < 0.05.*

**FIGURE 2:**
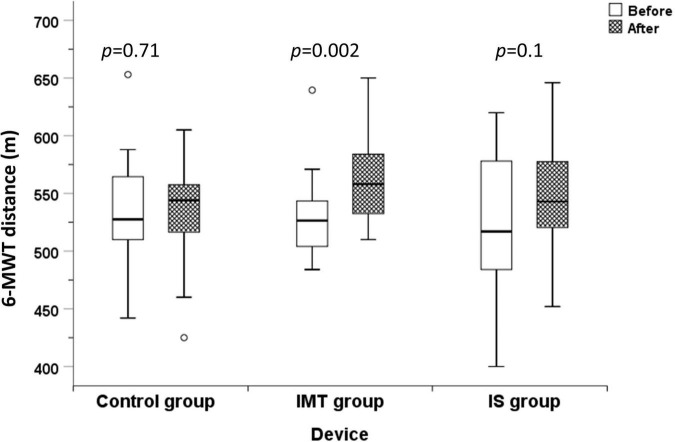
6-MWT distance before and after 8 weeks of training compared among the control, IMT, and IS group (6-MWT distance, 6-min walk test distance; m, meters; IMT, threshold inspiratory muscle training; IS, incentive spirometry).

**FIGURE 3:**
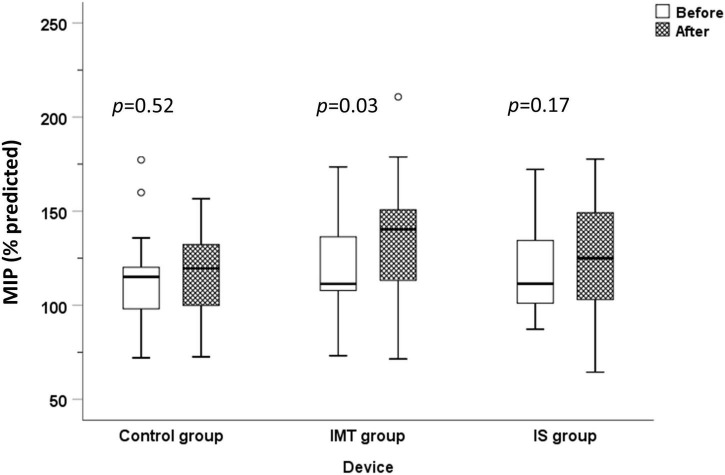
Maximum inspiratory pressure before and after 8 weeks of training compared among the control, IMT, and IS group (MIP, maximum inspiratory pressure; IMT, threshold inspiratory muscle training; IS, incentive spirometry).

Following 8 weeks of respiratory muscle training, significant increases in 6-MWT distance [25.0 (0.75, 74.0) m, *p* = 0.002] and MIP [9.33 (2.94, 15.7) cmH_2_O; *p* = 0.01, 9.40 (0.79, 13.0)%predicted; *p* = 0.03] were observed in the IMT group, but not in the IS or control groups (Table 2 and Figures 2, 3). However and importantly, there was a trend toward significant improvement in the 6-MWT distance in the IS group (*p* = 0.10). No significant differences were observed between before and after 8-weeks of respiratory muscle training in any of the 3 groups for the spirometric parameters FEV1, FVC, FEV1/FVC, FEF_25–75%_, and PEF. No significant difference among the IMT, IS, and control groups was found for any variable relative to change from baseline to after 8 weeks of respiratory muscle training (Table 2).

**TABLE 2:** Mean 6-min walk test distance, respiratory muscle strength, and spirometry before and after training compared among the control, IMT, and IS groups.

		Mean ± SD		
**Data**		**Control group (*n* = 20)**	**IMT group (*n* = 20)**	**IS group (*n* = 20)**
6-MWT distance (m)	Before	529.5 ± 52.2	528.5 ± 36.2	526.9 ± 59.1
	After	533.7 ± 44.0	561.5 ± 35.2	549.0 ± 50.6
	Difference	8.50 (-34.5,45.7)	25.0 (0.75,74.0)	14.5 (-25.5,47.2)
	*p*-value	0.71	***0.002***	0.1
MIP (cmH_2_O)	Before	98.6 ± 33.2	113.0 ± 27.8	109.3 ± 26.4
	After	101.4 ± 28.0	126.3 ± 25.9	114.4 ± 28.2
	Difference	3.16 (-3.78,15.2)	9.33 (2.94,15.7)	7.17 (-2.40,12.9)
	*p*-value	0.52	***0.01***	0.21
MIP (%Predicted)	Before	112.7 ± 26.1	121.2 ± 26.8	119.9 ± 25.2
	After	116.1 ± 23.7	135.3 ± 32.1	125.7 ± 30.6
	Difference	2.48 (-5.29,20.7)	9.40 (0.79,13.0)	5.25 (-2.52,15.8)
	*p*-value	0.52	***0.03***	0.17
FVC (%Predicted)	Before	101.7 ± 14.5	106.4 ± 12.6	114.6 ± 10.3
	After	100.2 ± 13.4	107.3 ± 13.8	114.0 ± 11.8
	Difference	-1.00 (-8.25,4.75)	0.50 (-3.25.3.75)	0.50 (-5.00,3.75)
	*p*-value	0.38	0.46	0.68
FEV_1_ (%Predicted)	Before	97.5 ± 16.8	100.1 ± 13.4	109.4 ± 11.2
	After	97.6 ± 15.8	101.2 ± 12.3	109.6 ± 13.8
	Difference	0.50 (-4.75,5.25)	0.50 (-2.00,3.75)	0.00 (-4.00,2.00)
	*p*-value	0.96	0.47	0.93
FEV_1_/FVC (%)	Before	85.0 ± 7.67	84.3 ± 6.89	85.6 ± 7.41
	After	86.4 ± 7.84	84.2 ± 5.66	85.9 ± 6.02
	Difference	1.41 (-2.03,3.67)	-0.47 (-1.78,0.48)	-0.92 (-2.46,1.52)
	*p*-value	0.16	0.89	0.82
FEF_25–75%_ (%Predicted)	Before	90.6 ± 27.3	86.3 ± 25.5	99.3 ± 28.1
	After	94.0 ± 27.6	87.7 ± 18.0	97.8 ± 23.7
	Difference	2.50 (-6.75,16.5)	-2.00 (-10.7,10.0)	-4.00 (-7.50,3.25)
	*p*-value	0.36	0.70	0.80
PEF (%Predicted)	Before	79.7 ± 21.3	78.2 ± 15.0	91.0 ± 19.8
	After	83.4 ± 23.1	77.6 ± 17.5	89.8 ± 17.9
	Difference	3.50 (-4.50,13.0)	0.50 (-11.2,8.50)	-1.00 (-10.5,11.2)
	*p*-value	0.21	0.80	0.81

*A p-value < 0.05 indicates statistical significance. IMT, threshold inspiratory muscle training; IS, incentive spirometry; 6-MWT distance, 6-min walk test distance; MIP, maximum inspiratory pressure; FVC, forced vital capacity; FEV1, forced expiratory volume in 1 s; FEF_25–75%_, forced expiratory flow rate within 25–75% of vital capacity; PEF, peak expiratory flow rate. Difference: median (IQR). The bold italic means a p-value < 0.05.*

## Discussion

The effects of threshold IMT on functional fitness and RMS have been investigated in adults with obesity, but not in children and adolescents with obesity. The present study is the first to study the effectiveness of threshold IMT on functional fitness, RMS, and spirometry, as well as the first RCT to compare the effects of threshold IMT and IS in obese children and adolescents. The results of this study demonstrated the benefit of an 8-week threshold IMT program for improving functional exercise capacity as measured by 6-MWT distance, and inspiratory muscle strength as measured by MIP in children and adolescents with obesity. The present study also observed a trend toward statistically significant improvement in the 6-MWT distance between before and after 8 weeks of respiratory muscle training in the IS group.

Several studies reported reduced RMS and functional fitness in children and adolescents with obesity (5–9). This is attributed to excessive fat deposition on the chest wall and abdomen, which contributes to dysfunction of the inspiratory muscles (especially the diaphragm), reduced chest wall compliance, reduced lung volume, and impaired lung mechanics that collectively lead to decreased functional fitness (8, 39).

The IMT device may benefit these patients by improving inspiratory muscle strength and endurance. Similar to our findings in children and adolescents, there have been many studies in adults that support the benefit of threshold IMT (15–17, 40) on inspiratory muscle strength and functional fitness. In contrast, studies of the IS device in obese adults are very limited. One study reported improved FEV_1_, FVC, and maximum voluntary ventilation after IS training, but RMS and functional fitness were not mentioned (19). The other study found significant improvement in 6-MWT distance after IS training (18). The mechanism of increased RMS after IMT may be multifactorial (12). The proposed mechanisms include increased proportion and size of type II muscle fibers (16, 41, 42), promoted diaphragm hypertrophy, attenuated respiratory muscle metaboreflex, and enhanced respiratory muscle economy. Improved RMS augments respiratory capacity, promotes muscle oxygenation, reduces lactate production by respiratory muscles, and eventually reduces respiratory muscle fatigue, which enhances functional fitness that facilitates exercise (18, 42, 43).

According to results of our study, the use of threshold IMT rather than IS may be suitable for improving the submaximal exercise capacity in obese children and adolescents. The explanation for the observed difference in outcome between these two devices is that the mechanism of threshold IMT is pressure-based loading, and the principle of use is inspiration against a set resistance pressure. This mechanism is like weight or resistance training exercise for inspiratory muscles, which is similar to weightlifting for strengthening extremity muscles. The main advantages of resistance training include improving muscle strength and endurance, enhancing the oxygen uptake of exercising muscles, and reducing muscle fatigue – all of which will improve functional fitness (12, 42, 43). By way of comparison, the IS device is a volume-based loading device. Its principle of use is inspiration to the TLC level, but the inspiration is not against resistance. The effectiveness of the IS for respiratory muscle training is, therefore, inferior to the threshold IMT, which leads to less improvement in functional fitness.

Threshold IMT is a portable, simple, user-friendly, and safe respiratory muscle training device for improving RMS and functional fitness. The results of this study and previous studies suggest that this device should be incorporated as an adjunctive therapeutic modality together with standard therapy of nutritional control and exercise in obese individuals. Threshold IMT might also benefit obese individuals who cannot tolerate exercise training, or who cannot perform outdoor exercise in some situations or settings. Further clinical trials to determine the appropriate protocol (frequency, duration, and resistance load) of threshold IMT in children and adolescents with obesity are needed. Previous RCTs in obese adults (15–17, 40) that reported substantial benefit after threshold IMT all commented on the importance of periodic adjustment of the training load to yield optimal training outcomes. Concerning other useful recommendations for improving respiratory muscles in clinical practice, Shei et al. (12) recommended that the training session be personalized, and to consider setting training goals, such as improving RMS and/or endurance, to facilitate longer training sessions.

### Limitations

This study has some mentionable limitations. First, even though our data was prospectively collected, the data included in this study was collected from a single center. Second, our threshold IMT intensity level might be low (40% of baseline MIP), and there was no adjustment of intensity level. Third, the threshold IMT device that was used in this study had a pressure level that ranged from 9 to 41 cm H_2_O; however, we used the only commercially available brand of threshold IMT device that is currently available in Thailand. Fourth and last, the 8-week duration of respiratory muscle training in this study may have been too short in this study population.

## Conclusion

Eight weeks of threshold IMT training significantly improved both inspiratory muscle strength (MIP) and functional capacity (6-MWT) in children and adolescents with obesity. Eight weeks of IS training yielded a trend toward significantly improved functional capacity. These results suggest that threshold IMT can be recommended as an adjunct therapy together with nutritional control and increased physical activity in obese children and adolescents. Based on the assumption that threshold IMT would help to reduce exercise intolerance, increased exercise would contribute to weight reduction, which is a main target of obesity management.

## Data Availability Statement

The raw data supporting the conclusions of this article will be made available by the authors, without undue reservation.

## Ethics Statement

The studies involving human participants were reviewed and approved by the Siriraj Institutional Review Board Faculty of Medicine Siriraj Hospital, Mahidol University Email: siiro@mahidol.ac.th. Written informed consent to participate in this study was provided by the participants’ legal guardian/next of kin.

## Author Contributions

KU contributed to the study conception, study design, statistical analysis, and manuscript preparation. PK recruited the study participants, conducted the fieldwork, performed the data collection, interpreted the data, and wrote the first draft of the manuscript. AN and PT contributed to the study conception and design. AP recruited the study participants and conducted the fieldwork. PC and KM conducted the fieldwork and performed the data collection. All authors contributed to the article and approved the submitted version.

## Conflict of Interest

The authors declare that the research was conducted in the absence of any commercial or financial relationships that could be construed as a potential conflict of interest.

## Publisher’s Note

All claims expressed in this article are solely those of the authors and do not necessarily represent those of their affiliated organizations, or those of the publisher, the editors and the reviewers. Any product that may be evaluated in this article, or claim that may be made by its manufacturer, is not guaranteed or endorsed by the publisher.
